# Specific Fertilization Practices Reveal Important Insights into the Complex Interaction Between Microbes and Enzymes in Soils of Different Farming Systems

**DOI:** 10.3390/life14121562

**Published:** 2024-11-28

**Authors:** Maša Pintarič, Ana Štuhec, Eva Tratnik, Tomaž Langerholc

**Affiliations:** Department of Microbiology, Biochemistry, Molecular Biology and Biotechnology, Faculty of Agriculture and Life Sciences, University of Maribor, Pivola 10, 2311 Hoče, Slovenia; ana.stuhec1@student.um.si (A.Š.); eva.tratnik1@student.um.si (E.T.); tomaz.langerholc@um.si (T.L.)

**Keywords:** agriculture, conventional–integrated, organic, biodynamic, fertilization, soil microorganisms, soil enzyme activity

## Abstract

The interaction of microorganisms and their enzyme activity is one of the key indicators for a comprehensive measurement of soil health. The aim of this study was to determine significant correlations between different soil microorganisms and enzyme activities of β-glucosidase, N-acetyl-glucosaminidase, urease, arylamidase, phosphatase, acid phosphatase, alkaline phosphatase, and arylsulfatase after supplementation with standard fertilizer, spent mushroom substrate and composed fertilizer in soils from conventional–integrated, organic and biodynamic farming. Samples were grouped according to the farming system and fertilization for all seasons. The biodynamic farm was the least affected by the different fertilizations, except for standard fertilization. Standard fertilizer caused negative correlations between the actinomycetes and the arylsulfatase in organic and biodynamic farms. The same fertilization affected the actinomycetes/phosphatase relationship differently, regardless of the basic soil structure. Actinomycetes correlated positively with acid phosphatase and urease in conventional–integrated and biodynamic farms after spent mushroom substrate, respectively. Arylamidase activity in relation to total microorganisms responded to fertilization with standard fertilizer and spent mushroom substrate independently of the basic soil structure. Fertilization can influence the soil microbe/enzyme relationships in different soils. Regardless of the basic soil structure, some of these relationships could be important indicators for further studies.

## 1. Introduction

The structure of soil microorganisms and their enzyme activity depends on the soil environment as well as the characteristics of the soil microorganisms themselves. Microorganisms and their activity can be influenced by biotic and abiotic factors [1,2]. They play a crucial role in providing a comprehensive measure of soil health. Microorganisms can swiftly respond to environmental changes, allowing them to adapt rapidly. This adaptability gives microbial analyses the potential to effectively assess soil health, with changes in microbial populations and enzyme activities serving as excellent indicators of shifts in soil health [3].

The soil microorganism community plays a vital role in maintaining healthy soil by facilitating essential biochemical processes such as organic matter decomposition and nutrient cycling. Additionally, these microorganisms contribute to plant growth by serving as natural fertilizers or pesticides, enhancing plant productivity in saline or alkaline soils, and providing support during periods of drought. Furthermore, soil microorganisms have shown promising results in remediating organic and heavy metal pollutants in the soil [4,5,6]. The biomass of microorganisms constitutes only a small proportion of soil organic matter, measuring less than 10%. Nonetheless, these microorganisms are abundant and exhibit significant taxonomic diversity [6]. Bacteria and fungi are the primary microorganisms found in soil, comprising 15% and 2% of the total living biomass, respectively [1,5,7]. Actinomycetes (class Actinomycetia [8]) are Gram-positive bacteria that belong to the phylum Actinomycetota (formerly Actinobacteria) [9]. The latest has garnered significant attention within the agricultural industry due to their function as biologically active compounds, plant growth promoters and regulators, and biocontrol agents [10,11]. In addition, the pharmaceutical and food industries utilize their secondary metabolites for various applications [12,13]. The prevalence of actinomycetes in soil generally exerts a positive influence on its structural composition [14]. One notable characteristic of these microorganisms is their ability to generate enzymes that break down complex organic substances in soil and sediments [15]. Fungi exhibit remarkable adaptive capabilities, enabling their growth across diverse environmental conditions [16,17]. Microorganisms of this nature serve a crucial function as biological regulators [18,19,20], playing a pivotal role in ecosystem management [16,21] and participating in the breakdown of organic matter and the transformation of compounds [22]. Yeasts and molds, as a unique type of fungi, may not be widely recognized as one of the most crucial functional groups in soil. Nonetheless, molds play a significant role in decomposing organic matter and promoting soil aggregation. Additionally, they contribute to reducing the pressure from soil-borne diseases [23,24,25]. There is still limited knowledge regarding the importance, function, and ecological interactions of yeasts in soil. However, there has been increasing focus on their potential to enhance plant growth in recent years. Yeasts have been identified as effective biostimulants, biofertilizers, and biocontrol agents against fungal pathogens. Additionally, they show promise as bioinsecticides, bioherbicides, and biodegraders, with potential for commercial applications as biofertilizers and biopesticides [26,27,28].

Enzymes play a crucial role in the assimilation of mineral and organic soil components. Consequently, they are intricately linked to soil physical properties, organic matter, microbial activity, and biomass [29]. The evaluation of soil quality and the regulation of plant decomposition often rely on the assessment of specific enzymes associated with various nutrient cycles [30]. β-Glucosidase (β-GLU), β-galactosidase, and N-acetyl-glucosaminidase (NAG) represent pivotal glucosidases responsible for catalyzing the hydrolysis of glycosides [30,31]. A positive correlation between glycosidases and soil pH, as well as organic carbon, has been reported [32]. Organisms with chitin in their cell walls, such as certain fungi, serve as the primary sources of N-acetyl-glucosaminidase (NAG). This enzyme not only participates in the carbon cycle but also performs a crucial function in the nitrogen cycle [33]. Furthermore, β-glucosidase (β-GLU) and its activity are known as the most commonly utilized soil quality indicator [32]. One of the key elements essential for plant growth is nitrogen, and its cycle is also influenced by the activity of urease (URE) and arylamidase (ARN). With the growing use of urea fertilization, URE has become a crucial factor in determining soil quality due to its pivotal role in controlling the availability of nitrogen for plants, which is essential for their growth and development [34,35]. The activity of ARN is closely linked to nitrogen mineralization as it facilitates the direct release of amino acids from organic matter [36,37,38]. The availability of phosphorus in the soil significantly impacts the initial growth of plants and is indispensable for all forms of life [39]. The presence of phosphatase (PHOS) activity can be used as an indication of the amount of inorganic phosphorus available in the soil for plants and microorganisms. It is a significant factor in determining soil quality [30]. The presence of this enzyme is closely linked to soil pH, resulting in a prevalence of acid phosphatases (PAC) in acidic soil and alkaline phosphatase (PAK) in basic soils [32]. Sulfur, as one of the essential elements necessary for the growth of living organisms, is intricately involved in numerous biochemical processes [40]. The mineralization of this element is crucial for enhancing its bioavailability in the soil, considering its predominantly organic existence [41]. One of the sulfatases, the arylsulfatase (ARS), plays a crucial role in the breakdown of organic sulfate esters [42,43]. This enzyme is primarily produced by fungi and bacteria, although some animals and lower plants are also known to produce it [40].

Soil fertilization represents one of the most ancient agricultural practices for enhancing soil fertility and regulating nutrient retention [1]. Notwithstanding, a wide array of diverse chemical fertilizers continues to be extensively and commonly overused, leading at times to adverse effects on the environment, as well as on human and animal health [1,4,44,45,46,47,48,49,50,51]. The utilization of spent mushroom substrate (SMS) as an organic fertilizer represents one of the key applications in agriculture [52], with its usage having seen a notable increase over the past decade. The composition of SMS includes proteins, carbohydrates, fats, and micronutrients present in a substantial amount of organic material, the proportions of which vary based on its fungal origin [53,54,55,56].

The activity of soil enzymes, which originates predominately from microbial sources, is closely intertwined with microbial activity. Consequently, alterations in their correlation resulting from various environmental interventions offer additional insights into the changes occurring in the soil. Therefore, the main goal of this research was to establish significant correlations between various soil microorganisms (total microorganisms, actinomycetes, and fungi) and soil enzymes (β-GLU, NAG, URE, ARN, ARS, PHOS, PAC, and PAK) following the application of different fertilizers in soils within conventional–integrated, biodynamic, and organic farming systems in Slovenia. As far as we know, our study is the first to examine these correlations, aiming to compare and evaluate their potential significance in various agricultural systems in Slovenia.

## 2. Materials and Methods

### 2.1. Location and Experimental Design

The research was conducted over the course of one year, from 2022 to 2023, and involved studying agricultural practices on three distinct farms (conventional–integrated (M), biodynamic (T), and organic (S)) in Northeastern Slovenia, Europe. The exact locations, sites, and characteristics of the three farms are described in Pintarič et al. [57]. Briefly, farm M was a conventional–integrated farm that is engaged in the integrated cultivation and processing of fresh seasonal vegetables and fruit. In Slovenia, the regulations on integrated production and the technological instructions for integrated production [58] define the production technology, control procedures, and type of labeling in integrated cultivation. These regulations are issued annually by the Ministry of Agriculture, Forestry, and Food [59]. Farm S is an organic-oriented farm and deals with the organic production of fresh seasonal vegetables and fruits. Organic farming in Slovenia adheres to the guidelines of Regulation (EU) 2018/848 of the European Parliament and the Council on organic production and labeling of organic products [60]. Farm T is a biodynamically oriented farm and is engaged in the organic production of fresh seasonal vegetables, fruit, and processed products according to the biodynamic method. Biodynamic agriculture in Slovenia adheres to the guidelines and standards set by the agriculture association, The Biodynamic Federation Demeter International [61].

The experimental plots were randomly laid out in triplicate (7.56 m^2^ for farm M, 5.88 m^2^ for farm S, and 2.94 m^2^ for farm T), and fertilization was carefully administered over a period of two years during the spring, following the specific guidelines of the experimental design:

Soil without fertilizer (C: control);
Soil with composted spent mushroom substrate (SMS);
Soil with standard fertilization (SF);
Soil with SF and SMS in a 50/50 ratio (SF.SMS).

The fertilization in a single dose of the basic plots was standardized according to the nitrogen content on all participating farms. The nitrogen content was calculated for each farm based on the results of the soil analysis, the SMS, and the SF. The amount of SMS added to the soil was adjusted to be equivalent in terms of the nitrogen content to that of the SF treatment. SF was applied according to common practice, specific for each farm: the basic fertilizer in farm M was organic fertilizer “Bioorganik” (a single dose of 500 kg/ha), and in farm S, it was pelleted manure (commercial fertilizer for organic farming “Fertorganico”—a single dose of 600 kg/ha and “Patent K”—single dose of 800–1200 kg/ha)) and in farm T was a compost/sunflower seed cake (own fertilizer—a single dose of 2 kg/m^2^ compost and of 1 kg/m^2^ sunflower seed cake every second year). On farm M, the mineral fertilizer “Rosasol K” was also applied (1 kg/1000 L water/ha weekly during the growing season). The composition of fertilizers, as well as their N:P:K ratios, are shown in Supplementary Tables S1–S4. The chemical properties of SMS and soil properties of the three farms are shown in Supplementary Table S5. A detailed composition of the solid mushroom substrate and its composted technique are described in [57]. During the growth season, tomatoes were cultivated in all experimental fields.

### 2.2. Analysis of Soil Microorganisms and Enzymes

#### 2.2.1. Soil Sampling

After two years of fertilization, soil samples were taken to analyze the number of microorganisms and the enzyme activity. Sampling was conducted during each season, including winter (January), spring (April), summer (July), and autumn (October). Please refer to the detailed description of soil sampling in [57].

#### 2.2.2. Determination of Soil Moisture

The gravimetric soil moisture content (%) was determined as described in Brooks [62], and a detailed protocol can be found in Pintarič et al. [57].

#### 2.2.3. Sampling for Quantitative Microorganism Analysis

The standard serial dilution method and spreading technique on agar plates were used to perform the quantitative analysis of the total number of microorganisms, actinomycetes, and fungi. The results were quantified as the number of colonies per gram of dry sample [CFU/g of dry sample], taking into account the content of gravimetric moisture. Please refer to the detailed protocol in Pintarič et al. [57].

#### 2.2.4. Sampling for Enzyme Analysis

The samples underwent processing in accordance with the guidelines outlined in the ISO standard 20130:2018 [63]. The enzyme activity was quantified and expressed as [mU/g] of dry sample [nmol/min/g of dry sample]. The enzyme activity measured in this study is listed in Table 1.

### 2.3. Statistical Analysis of Microorganism Quantities and Enzyme Activity

Samples were grouped according to the farm (M, T, and S) and fertilization treatment (C, SF, SMS, and SF.SMS). Results from microorganism quantities and enzyme activity from 12 groups were statistically correlated for all four seasons (winter, spring, summer, and autumn).

The data we collected were thoroughly examined and processed using SPSS IBM Statistics 29.0 (IBM Inc., Armonk, NY, USA) for analysis. Association analysis between two continuous variables was performed using the Pearson product–moment correlation or Spearman correlation after the Shapiro–Wilk test for normality. The significance level (α) was established at 5%, and any *p*-value less than 0.05 was deemed to be statistically significant.

## 3. Results

### 3.1. Correlation Rates in the Control Group During Seasonal Variations

**Figure 1:**
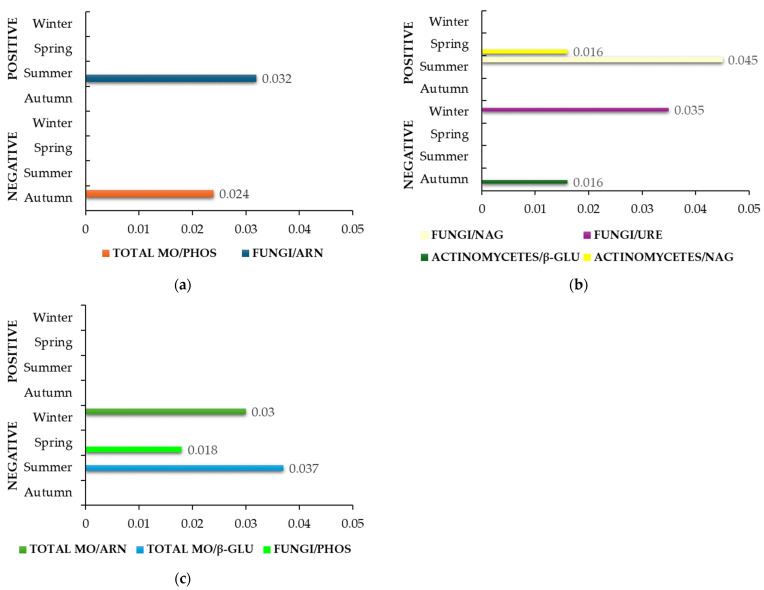
Statistically significant correlation factors (*p* < 0.05) in the C group (soil without fertilizer) during seasonal variations. (**a**) Farm M; (**b**) Farm T; (**c**) Farm S. Microorganisms (MO); arylamidase (ARN); β-glucosidase (β-GLU); N-acetyl-glucosaminidase (NAG); phosphatase (PHOS); urease (URE). The lack of bars in a specific season indicates that no statistically significant correlations were identified.

Significant correlations in the control (C) group for all three farms are shown in Figure 1. Farm M (Figure 1a) showed a strong positive association and statistical significance (ρ = 0.999, *p* = 0.032) between fungi and ARN. A strong negative association and a statistical significance (ρ = 0.999, *p* = 0.024) were observed between the total number of microorganisms and PHOS. Figure 1b shows the correlation rates in farm T, where NAG positively correlated to fungi (ρ = 1.000, *p* = 0.016) in summer and actinomycetes (ρ = 0.997, *p* = 0.045) in spring. However, actinomycetes negatively correlated with β-GLU (ρ = 1.000, *p* = 0.016) in autumn. In winter, a negative association and statistical significance were observed between fungi and URE (ρ = 0.998, *p* = 0.035). Only negative correlations were observed in farm S (Figure 1c). Total microorganisms count negatively correlated to ARN (ρ = 0.999, *p* = 0.030) in winter and to β-GLU (ρ = 0.998, *p* = 0.037) in summer. A negative correlation between fungi and PHOS (ρ = 1.000, *p* = 0.018) was also found in spring.

### 3.2. Effect of SF on Correlation Rates During Seasonal Variations

**Figure 2:**
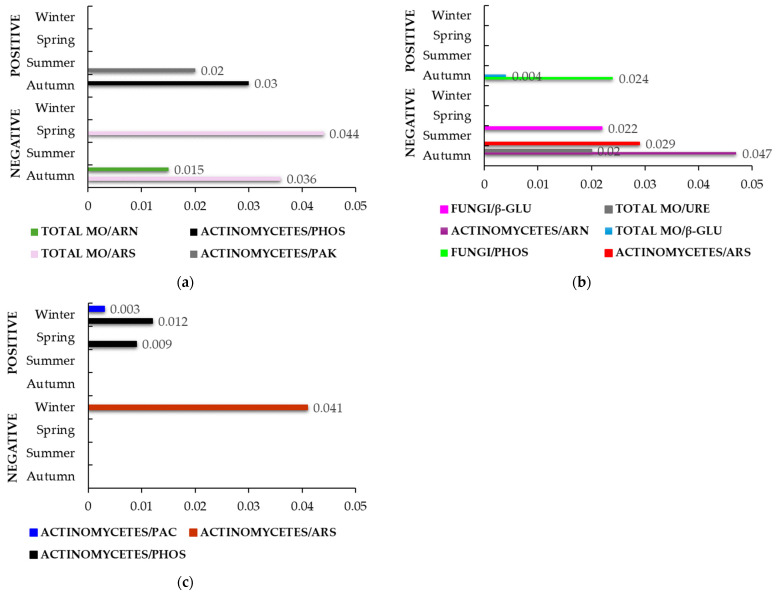
Statistically significant correlation factors (*p* < 0.05) in the SF group (soil with standard fertilization) during seasonal variations. (**a**) Farm M; (**b**) Farm T; (**c**) Farm S. Microorganisms (MO); arylamidase (ARN); arylsulfatase (ARS); β-glucosidase (β-GLU); phosphatase (PHOS); acid phosphatase (PAC); alkaline phosphatase (PAK); urease (URE). The lack of bars in a specific season indicates that no statistically significant correlations were identified.

Figure 2 shows statistically significant correlation rates in the SF group of all three farms. After the addition of SF in farm M (Figure 2a), actinomycetes correlated positively with PAH (ρ = 0.999, *p* = 0.020) in summer and with PHOS (ρ = 0.999, *p* = 0.030) in autumn. However, a strong negative correlation and statistical significance was found between the total number of microorganisms and ARS in spring (ρ = 0.998, *p* = 0.044) and in autumn (ρ = 0.998, *p* = 0.036). In addition, the total number of microorganisms also correlated negatively with ARN in the autumn (ρ = 0.998, *p* = 0.015). In farm T, several correlation rates of all three groups of microorganisms were detected in summer and autumn due to SF application (Figure 2b). In the autumn, the total number of microorganisms had a strong positive association and high statistical significance with β-GLU (ρ = 1.000, *p* = 0.004), but a negative correlation with URE (ρ = 0.999, *p* = 0.020) in the summer. Actinomycetes had a negative correlation with ARS (ρ = 0.999, *p* = 0.029) in summer and with ARN (ρ = 0.997, *p* = 0.047) in autumn. Fungi had a positive correlation with PHOS (ρ = 0.999, *p* = 0.024) in the autumn and a negative correlation with β-GLU (ρ = 0.999, *p* = 0.022) in the summer. In farm S, the application of SF only affected the correlation rates between actinomycetes and some enzymes (Figure 2c). Interestingly, the correlations were only found in winter and spring. In winter, actinomycetes correlated positively with PAC (ρ = 1.000, *p* = 0.003) and PHOS (ρ = 1.000, *p* = 0.012) but negatively with ARS (ρ = 0.998, *p* = 0.041). A strong positive correlation and high statistical significance were found between actinomycetes and PHOS in spring (ρ = 1.000, *p* = 0.009).

### 3.3. Effect of SMS on Correlation Rates During Seasonal Variations

**Figure 3:**
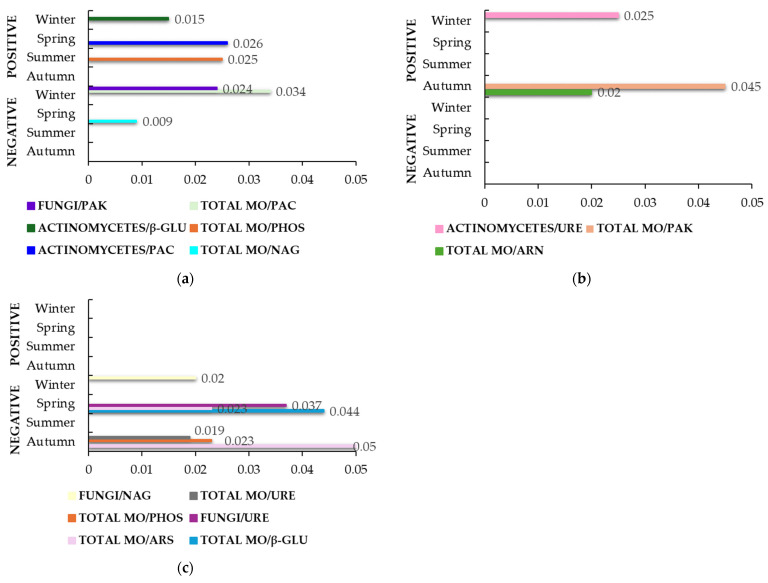
Statistically significant correlation factors (*p* < 0.05) in the SMS group (soil with composted spent mushroom substrate) during seasonal variations. (**a**) Farm M; (**b**) Farm T; (**c**) Farm S. Microorganisms (MO); arylamidase (ARN); arylsulfatase (ARS); β-glucosidase (β-GLU); N-acetyl-glucosaminidase (NAG); phosphatase (PHOS); acid phosphatase (PAC); alkaline phosphatase (PAK); urease (URE). The lack of bars in a specific season indicates that no statistically significant correlations were identified.

Figure 3 shows the variations in correlation rates after fertilization with SMS. During the seasonal changes, the total number of microorganisms correlated most strongly in farm M (Figure 3a). Indeed, there was a positive correlation with PHOS (ρ = 0.999, *p* = 0.025) in summer and a negative correlation with PAC (ρ = 0.999, *p* = 0.034) in winter, followed by a strong negative association and high statistical significance with NAG (ρ = 1.000, *p* = 0.009) in spring. In addition, a positive correlation was found between actinomycetes and β-GLU (ρ = 1.000, *p* = 0.015) in winter, followed by PAC (ρ = 0.999, *p* = 0.026) in spring. However, fungi correlated negatively with PAK (ρ = 0.999, *p* = 0.024) in winter. The SMS application revealed only a few correlations related to winter and autumn in farm T (Figure 3b). A positive correlation between actinomycetes and URE (ρ = 0.999, *p* = 0.025) was found in winter. Finally, in autumn, the total number of microorganisms correlated positively with PAK (ρ = 0.998, *p* = 0.045) and ARN (ρ = 0.999, *p* = 0.020). Figure 3c shows the correlation rates in farm S. Fertilization with SMS appeared to influence the number of fungi and total microorganisms, resulting in several negative correlations. Fungi correlated negatively with NAG (ρ = 1.000, *p* = 0.020) in winter, followed by URE (ρ = 0.998, *p* = 0.037) in spring. In the same season, the total number of microorganisms correlated negatively with ARS (ρ = 0.999, *p* = 0.023) and β-GLU (ρ = 0.998, *p* = 0.044). In addition, a negative correlation between the total number of microorganisms and URE (ρ = 1.000, *p* = 0.019), PHOS (ρ = 0.999, *p* = 0.023), and ARS (ρ = 0.997, *p* = 0.050) was revealed in autumn.

### 3.4. Effect of SF.SMS on Correlation Rates During Seasonal Variations

**Figure 4:**
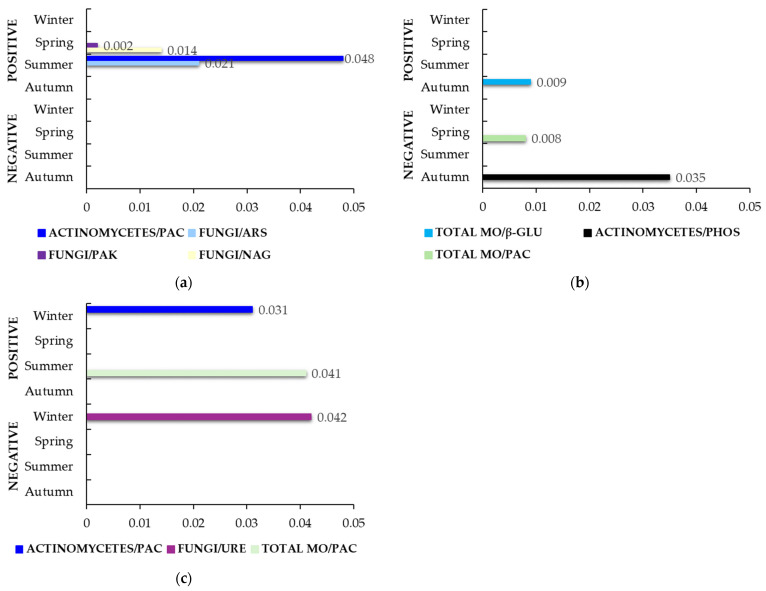
Statistically significant correlation factors (*p* < 0.05) in the SF.SMS group (soil with SF and SMS in a 50/50 ratio) during seasonal variations. (**a**) Farm M; (**b**) Farm T; (**c**) Farm S. Microorganisms (MO); arylsulfatase (ARS); β-glucosidase (β-GLU); N-acetyl-glucosaminidase (NAG); phosphatase (PHOS); acid phosphatase (PAC); alkaline phosphatase (PAK); urease (URE). The lack of bars in a specific season indicates that no statistically significant correlations were identified.

A combination of SF and SMS fertilizers (SF.SMS) and their effect on the correlations in three farms is shown in Figure 4. Farm M (Figure 4a) showed only positive correlations. In spring, a strong positive correlation and high statistical significance were found between fungi and PAK (ρ = 1.000, *p* = 0.002). In addition, fungi correlated positively with NAG (ρ = 1.000, *p* = 0.014) in the same season, followed by ARS (ρ = 0.999, *p* = 0.021) in summer. During the summer season, a notable positive correlation was observed between actinomycetes and PAC (ρ = 0.997, *p* = 0.048). A combined fertilizer in farm T (Figure 4b) showed a strong negative association and a high statistical difference between the total number of microorganisms and PAC in spring (ρ = 1.000, *p* = 0.008). In addition, in autumn, the total microorganisms count correlated negatively with β-GLU (ρ = 1.000, *p* = 0.009), and actinomycetes correlated negatively with PHOS (ρ = 0.998, *p* = 0.035). In farm S (Figure 4c), actinomycetes had a positive correlation with PAC (ρ = 0.999, *p* = 0.031), while fungi had a negative correlation with URE (ρ = 0.998, *p* = 0.042) in winter. Overall, a positive correlation was found between the total number of microorganisms and PAC (ρ = 0.998, *p* = 0.041).

## 4. Discussion

Farm M was found to have the least microbiologically and enzymatically active soil compared to the other two farms. This could be attributed to the relatively low number of microorganisms and the low activity of enzymes present in the soil [57]. These results reflected the conventional–integrated farming method, the use of mineral fertilizers, and the soil structure (lower organic matter and total carbon, as well as lower moisture content, reflected lower microbial diversity, abundance, and enzyme activity—Supplementary Table S5). It is, therefore, not surprising that in the C group, only two correlations between microorganisms and enzymes were found in farm M (Figure 1a), compared to the same group in farms T (Figure 1b) and S (Figure 1c). In fact, the number and the activity of microorganisms were also higher in both farms [57]. Based on the positive correlation between the number of fungi and ARN in summer in farm M (Figure 1a), we could assume that fungi were the main microorganisms responsible for the high ARN activity in this season within this group. Relatively low content of moisture (Supplementary Table S5) in the soil of farm M could also favor the growth of fungi rather than bacteria [16,17]. Interestingly, bacteria are the predominant producers of this enzyme, followed by yeasts and some other fungi [64,65,66,67,68]. In addition, a negative correlation between the activity of PHOS and the total number of microorganisms was observed in autumn. This result could be a consequence of the available non-organic phosphorus in the soil and, therefore, not directly related to the microorganism enzyme in question. The high activity of the biodynamic soils and its high content of organic carbon (Supplementary Table S5) led to positive correlations between the activity of NAG and the fungi/actinomycetes (Figure 1b), confirming previous studies linking fungi [33] and actinomycetes [15] as NAG producers that also degrade complex organic substances typical of biodynamic agriculture [69,70]. Interestingly, due to the negative correlation with fungi, the activity of URE in winter appears to be of bacterial or plant rather than fungal origin [71]. Moreover, the high activity of β-GLU in autumn in farm T [57] seems to originate from fungi, other bacteria, plants, and/or animals [72] rather than from actinomycetes. In fact, our results (Figure 1b) showed a negative correlation between the β-GLU activity and the actinomycetes. The soil without fertilizer (group C) in organic farm S (Figure 1c) showed less correlations than farm T (Figure 1b) but more than farm M (Figure 1a). However, negative correlations between the total number of microorganisms and ARN (Figure 1c) were observed in winter (Figure 1c), which is interesting as ARN showed very low activity [57] in this season. That could possibly be due to excessively low temperatures [38] and other abiotic, biotic, and anthropogenic factors [73] that could influence its activity during this season more than microorganisms themselves. In fact, the number of microorganisms was very low as well [57]. In addition, the activity of β-GLU correlated negatively with the total number of microorganisms in summer. Therefore, the lower number of microorganisms found in summer [57] could not be directly related to the lower β-GLU activity in the same season, except for the anaerobic microorganisms. In fact, microorganisms with high cellulolytic activity, such as anaerobic fungi, are β-GLU producers as well [74]. Additionally, the β-GLU activity could indeed originate from anaerobes as the total number of microorganisms on tryptic soy agar (TSA) was assessed after aerobic incubation [57]. Further, the activity of PHOS was quite high in spring in farm S. This may be due to the activity of PAC rather than PAK, as the soil in this farm is quite acidic (Supplementary Table S5). Furthermore, the activity of PHOS depends on the amount and diversity of the microbial composition, as well as on the organic composition of the soil [69,75], which was high, typical for organic soil in farm S (Supplementary Table S5). However, the activity of PHOS was negatively correlated with the number of fungi (Figure 1c), which was rather low in spring [57]. These results could point out the possibility of allowing other microorganisms, such as phosphatase-producing bacteria, to grow and, therefore, be primarily responsible for the amount of available phosphorus in the soil [76,77,78].

Supplementation with SF revealed some new correlations between microorganisms and enzymes (Figure 2). In farm M (Figure 2a), actinomycetes correlated positively and strongly with PHOS and PAK in autumn and summer, respectively. In fact, the increase in PHOS activity during autumn, following the application of SF, may be linked to a surge in the actinomycetes population during the same season, just as the decrease in PAK activity in the summer could be attributed to the reduction in the number of actinomycetes in the same season [57]. Although actinomycetes are primarily known to be involved in the nitrogen cycle [10,14], there are reports showing their potential, but not yet fully understood, role as producers of acid/alkaline phosphatases that play a part in the hydrolysis of organic phosphorylated compounds [79,80]. In farm M (Figure 2a), the activity of ARS (in autumn and spring) and ARN (in autumn) was negatively correlated with the total number of microorganisms. The supplementation of SF reduced the activity of both enzymes in the mentioned seasons [57], which could be a consequence of the presence of other microorganisms whose activity could be increased in the same seasons. In addition, it has been reported that mineral fertilizers have an inhibitory effect on ARS activity ([73] and Supplementary Table S2). Supplementation with SF also led to few negative correlations in farm T (Figure 2b). The unexplained increase in ARS activity in summer could be related to the lower amount of actinomycetes in the same season [57] due to their negative association with actinomycetes in the same season (Figure 2b) and the fact that this enzyme is mainly produced by fungi in addition to certain bacteria, lower plants, and animals [40]. Alternatively, these results could be explained by a simple competition of microbial growth leading to reduced growth of actinomycetes and increased growth of other ARS-producing microorganisms. However, some other negative correlations emerged during the summer in farm T (Figure 2b), of which the negative association between the total number of microorganisms and URE showed that URE activity may be of plant or animal origin rather than microorganism origin. URE is a widely distributed enzyme known to be present in many organisms and different environments [71]. In the same season, there was also a slight increase in β-GLU activity after the addition of SF in farm T [57], but its activity was negatively correlated with the fungi (Figure 2b), ruling out the possibility that cellulase-producing fungi were responsible for the increase in summer. On the contrary, β-GLU activity correlated positively with the total number of microorganisms in autumn (Figure 2b). However, supplementation with SF had the smallest effect on the number of microorganisms and β-GLU activity compared to other fertilizations [57]. In the same season in farm T, SF had the strongest influence on the number of fungi of all fertilization types [57], which led to a positive correlation with PHOS activity (Figure 2b). Although the non-optimal low temperature in winter caused low PHOS and PAC activity and a low number of actinomycetes in farm S [57], a positive correlation between the above parameters was observed after the addition of SF (Figure 2c). This fact could again confirm a possible, perhaps even important, role of actinomycetes in the phosphorus cycle [79,80]. Moreover, a positive correlation between PHOS and actinomycetes after SF addition was also observed in spring (Figure 2c). With the increase in temperature and the warming of the weather in spring, the number of actinomycetes and the activity of PHOS also increased [57]. Presumably, the increased activity of PHOS was due to the increase in PAC rather than PAK, as indicated by the acidic soil condition in farm S (Supplementary Table S5). Interestingly, fertilization with SF in winter in farm S led to a negative correlation between actinomycetes and ARS (Figure 2c), a correlation that was also observed in farm T after the same fertilization but in summer (Figure 2b). However, in this case, the number of actinomycetes and the activity of ARS was quite low [57].

The addition of SMS fertilizer in farm M significantly increased the activity of PAC in spring [57], which positively correlated with actinomycetes in the same season (Figure 3a), again confirming a link to phosphatase-producing actinomycetes [79,80]. As previously mentioned, actinomycetes are known as organic decomposers, and their increase after SMS addition in winter could influence the increase in β-GLU activity in the same season [57], as a strong positive correlation between the two parameters was found (Figure 3a). Additionally, it is known that β-GLU is one of the cellulolytic enzymes also produced by cellulase-producing actinomycetes [81]. Furthermore, the addition of SMS increased the total number of microorganisms in summer in farm M [57], and this could be directly related to the positive correlation found between the activity of PHOS and the total number of microorganisms in the same season (Figure 3a). Indeed, the activity of PHOS varies according to the quantity and diversity of soil microbial composition and organic matter [82], with the SMS content being abundant (Supplementary Table S5). The addition of SMS also revealed some negative correlations in farm M (Figure 3a). In spring, the total number of microorganisms correlated negatively with the activity of NAG, as it did with PAC in winter. As NAG became inactive in spring, its inhibition could be related to a significantly increased activity of some microorganisms and their enzymes added with SMS [57]. Furthermore, fertilization with SMS led to a slight increase in the fungal number in winter [57], which was negatively correlated with PAK in the same season (Figure 3a). It has been reported that the reduction in the activity of PHOS is directly related to the increase in available phosphorus in the soil [83]. PAK mainly catalyzes the hydrolysis of phospholipids, resulting in the release of inorganic phosphorus [77]. However, the availability of phosphorus also depends, among other things, on the solubilization of inorganic phosphate by many free-living and symbiotic fungi [76], a fact that can confirm the above results. Unlike in farm M, the SMS addition in farm T showed only a few positive correlations (Figure 3b), indicating the balanced ratio in the active biodynamic soil, independent of the addition of any kind of fertilization. The number of actinomycetes correlated positively with URE, suggesting the involvement of this enzyme in the nitrogen cycle and its regulation by the mentioned association. Further studies should focus on this, as this association was found in winter, where another enzyme—ARN, which is also important for the nitrogen cycle, showed less activity [57]. Further, the total number of microorganisms correlated positively with ARN and PAK in the autumn in farm T (Figure 3b), while a significant decrease in the total microorganisms count after the addition of SMS was observed as well [57]. This could be directly related to the decrease in ARN activity but not PAK activity [57]. In fact, the activity of PAK was increased after SMS [57] and, therefore, could be related to other organisms in the soil and/or pH [30,32]. Supplementation with SMS resulted in quite a few negative correlations between fungi, total microorganisms, and various enzymes in farm S (Figure 3c). There was a statistically significant increase in fungi after SMS in winter in farm S [57]; however, fungi correlated negatively with NAG activity in the same season (Figure 3c). Although NAG activity was quite low in winter [57], it seems that the production of this enzyme, in this case, is related to other microorganisms, most likely actinomycetes [15] or other NAG-producing bacteria. A similar explanation could be given for the negative correlation between fungi and URE in spring (Figure 3c). Total microorganisms correlated negatively with β-GLU and ARS in spring. The higher production and activity of these two enzymes after the addition of SMS in spring [57] suggests that they may be involved in the higher decomposition of organic matter and carbon cycle by SMS. However, it appears that the overall activity of the microorganisms does not control their activity and function due to their negative association (Figure 3c) but could be related to other biotic parameters [73,84]. Finally, a negative correlation was also found between the total microbial count and ARS, URE, and PHOS in the autumn (Figure 3c). Since the increased number of total microorganisms in this season after SMS in farm S [57] could be a result of an increase in actual soil microorganisms or microorganisms supplemented in SMS or both, these negative correlations could be due to the already enzymatically active SMS.

Interestingly, the addition of combined fertilizer (SF.SMS) in farm M (Figure 4a) resulted in positive correlations between fungi and NAG/PAK, confirming the role of fungi as the main producers of NAG [33] and PAK in spring, which could be due to the increased demand for nitrogen and phosphorus, respectively, in the soil during this season. In summer, ARS activity was increased after the addition of the combined fertilizer [57], probably due to the partial decomposition of organic matter from SMS [85]. However, its activity was positively correlated with the number of fungi (Figure 4a), confirming the fact that many microorganisms, including fungi, are responsible for ARS production [86]. In addition, actinomycetes could, in turn, be associated with phosphatase-producing microorganisms [79,80], as we found that they were positively correlated with PAC in summer (Figure 4a). We also found this correlation after the addition of SMS (albeit in spring) (Figure 3a), indicating a direct relationship between SMS/actinomycetes/PAC in the soil of farm M. A similar pattern, but after different fertilizer effect, was observed in farm T (Figure 4b). Supplementation with SF.SMS fertilizer again showed a strong positive association and high significance between the number of total microorganisms and β-GLU in autumn, which may be due to SF fertilizer rather than SMS, as the same correlation was observed after the addition of SF alone in the same season (Figure 2b). In fact, fertilization with SF and its addition in the combined fertilizer decreased the number of total microorganisms as well as the activity of β-GLU compared to the C group [57] in farm T, indicating the importance of bacteria/fungi/β-GLU in relation to the carbon cycle, its utilization and its abundance in the soil [87]. Furthermore, the addition of SF.SMS in farm T resulted in two new negative correlations (Figure 4b). Actinomycetes correlated negatively with the activity of PHOS, a relationship that can be confirmed and explained by a higher number of actinomycetes and a decrease in PHOS activity in the combined group compared to group C [57]. Furthermore, in spring, a negative correlation between total microorganisms and PAC activity was observed (Figure 4b). Since total microorganisms included all aerobic bacteria, including fungi [57], and the addition of combined fertilizer resulted in a decrease in the number of actinomycetes, fungi, and PAC activity compared to the C group [57], the observed correlation could be related to other bacteria not determined in this study. In farm S, supplementation with a combined fertilizer had a significant effect on only three relationships (Figure 4c), compared to supplementation with SF (Figure 2c) and SMS (Figure 3c) alone. In winter, actinomycetes again correlated positively with PAC (Figure 4c), most likely due to the influence of SF in this fertilizer, as we found the same strong positive correlation between the two parameters after supplementation with SF alone (Figure 2c). The activity of PAC also correlated positively in summer, but with total microorganisms (Figure 4c), which, as mentioned above, comprise a large group of microorganisms consisting of PAC-producing bacteria, actinomyces, and PAC-producing fungi as well. Moreover, fungi and URE correlated negatively in winter (Figure 4c), a relationship that was also observed after supplementation with SMS (Figure 3c), although not in the same season. Nevertheless, the same correlation could be due to the addition of SMS rather than SF in the combined fertilizer.

## 5. Conclusions

Different correlations between microorganisms and enzymes in the control group showed the diversity of soils in three farms and the importance of their relationship. Apart from SF, supplementation with SMS and SF.SMS had no drastic impact on correlation rates in the biodynamic farm, demonstrating the important role of a balanced relationship between biotic and abiotic factors in soil function and health. However, this was not the case in conventional–integrated and organic soils, where SMS alone appeared to affect many correlations between total microorganisms and the various enzyme activities, as most of them were not affected after supplementation with composed SF.SMS fertilizer. Supplementation with SF alone seemed to cause a greater imbalance, especially between actinomycetes and some enzymes, in all farms compared to the control. Actinomycetes correlated negatively with ARS after SF in organic and biodynamic farms, suggesting that the addition of SF has an influence on ARS activity, mainly originating from fungi or other bacteria in soils with higher abundance and diversity. Moreover, an interesting observation was made in relation to PHOS. Regardless of the basic soil structure, actinomycetes correlated differently with PHOS after supplementation with SF (conventional–integrated farm and organic farm), SMS (conventional–integrated farm), or SF.SMS (biodynamic farm). This clearly indicates a connection between this enzyme and actinomycetes due to different fertilization, regardless of the basic soil structure, a fact that has not yet been clearly investigated. Furthermore, supplementation with SMS clearly had a positive effect on the relationship between actinomycetes and PAC in the conventional–integrated farm, as the same correlations were also observed after the addition of SF.SMS. Additionally, actinomycetes were positively related to the activity of URE, and this seemed to be of utmost importance for nitrogen cycling during winter in the biodynamic farm. Finally, our results showed that the relationship between total microorganisms and ARN might respond to fertilization with SF or SMS regardless of the basic soil structure.

Our study has shown that fertilization can influence the relationship between soil microbes and soil enzyme activity in different soils. However, some of these relationships were independent of the basic soil structure and could be important indicators for further studies.

## Figures and Tables

**Table 1:** Enzyme activity measurements with colorimetric method.

Enzyme	Abbreviation	EC Number
Arylamidase	ARN	E.C. 3.4.11.2
Arylsulfatase	ARS	E.C. 3.1.6.1
β-Glucosidase	β-GLU	E.C. 3.2.1.21
N-acetyl-glucosaminidase	NAG	E.C. 3.2.1.52
Phosphatase	PHOS	E.C. 3.1.4.1
Acid phosphatase	PAC	E.C. 3.1.4.1
Alkaline phosphatase	PAK	E.C. 3.1.4.1
Urease	URE	E.C. 3.5.1.5

EC: Enzyme Commission number.

## Data Availability

The raw data supporting the conclusions of this article will be made available by the authors upon request.

## Supplementary Materials

The following supporting information can be downloaded at: https://www.mdpi.com/article/10.3390/life14121562/s1, Table S1: Organic fertilizer “Bioorganik”, Table S2: Mineral fertilizer “Rosasol K”, Table S3: Organic fertilizer “Fertorganico”, Table S4: Organic mineral fertilizer “Patent K”, Table S5: Initial properties of the SMS and the soils.
