# Supplementary material for: Specific Fertilization Practices Reveal Important Insights into the Complex Interaction Between Microbes and Enzymes in Soils of Different Farming Systems
Source: Life (Basel). 2024 Nov 28;14(12):1562. doi: 10.3390/life14121562 (PMC11676776; doi:10.3390/life14121562)
Supplement: Supplementary file 1 [file life-14-01562-s001.zip › Supplementary tables.pdf]

Table S1. Organic fertilizer "Bioorganik".

Nutrient	Nutrient content (%)	N/P/K ratio
Total N	5	3.5
P	0.1	2.3
K	0.02	0.2
Ca	16	
Total Mg	2	
B	0.1	
Cu	0.02	
Fe	1.5	
Mn	0.02	
Zn	0.12	

Table S2. Mineral fertilizer "Rosasol K".

Nutrient	Nutrient content	N/P/K ratio
Total N	12 %	12
P	12 %	12
K	36 %	36
B	100 ppm	
Cu	75 ppm	
Fe	260 ppm	
Mn	320 ppm	
Zn	230 ppm	

Table S3. Organic fertilizer "Fertorganico".

Nutrient	Nutrient content (%)	N/P/K ratio
Total N	11	11
P	0	0
K	0	0

Table S4. Organic mineral fertilizer "Patent K".

Nutrient	Nutrient content (%)
K	24,9
Mg	6
S	17,6

Table S5. Initial properties of the spent mushroom substrate (SMS) and the soils.

Parameter	SMS	Farm M	Farm S	Farm T
pH	7.6	7	4.2	7.1
Moisture	84.4	17.1	28.1	26.3
P_2O_5 (mg/100 g)	345	21	19.5	23
K_2O (mg/100 g)	2015	12	17.5	30.5
Mg (mg/100 g)	102	32	14	37.5
Ca (mg/100 g)	987.5	446	48	557
Total N (%)	1.52	0.16	0.20	0.31
N-NH^+_4 (mg/kg)	48	1.2	0.7	0.5
N-NO^-_3 (mg/kg)	0.5	144.6	1.0	60.6
Total organic C (%)	32.4	1.6	2.1	3.4
Organic matter (%)	58	2.7	3.5	5.9
